# Characterization of *Dendrolimus houi* Lajonquiere (Lepidoptera: Lasiocampidae) Transcriptome across All Life Stages

**DOI:** 10.3390/insects10120442

**Published:** 2019-12-09

**Authors:** Xiaohong Han, Ciding Lu, Scott M. Geib, Junxian Zheng, Songqing Wu, Feiping Zhang, Guanghong Liang

**Affiliations:** 1 Forestry College, Fujian Agriculture and Forestry University, Fuzhou 350002, China; hxhdax@163.com (X.H.); dabinyang@126.com (S.W.); 2 Daniel K. Inouye US Pacific Basin Agricultural Research Center, USDA-ARS, 64 Nowelo, St.; Hilo, HI 96720, USA; scott.geib@usda.gov; 3 Provincial Key Laboratory of Integrated Pest Management in Ecological Forests, Fujian Agriculture and Forestry University, Fuzhou 350002, China; fpzhang1@163.com

**Keywords:** *Dendrolimus houi* Lajonquiere, transcriptome, high throughput sequencing, developmental stage, differential gene expression

## Abstract

*Dendrolimus houi* Lajonquiere is a phytophagous caterpillar infesting many economically important coniferous tree species in China, causing serious economic and ecological environment losses. Based on previous research, it has one generation per year in South China and East China in contrast to two generations per year in Yunnan province in southwestern China. The species is potentially resilient to climatic extremes in these regions with the eggs and 1st instar larvae surviving in the winter (5 °C), older instar larvae and pupae surviving high temperatures in the summer (35 °C), suggesting some temperature stress tolerance during different developmental stages. However, little is known in this species at the genetic and genomic level. In this study, we used high throughput sequencing to obtain transcriptome data from different developmental stages (eggs, 1st–3rd instar larvae, 4th–5th instar larvae, 6th–7th instar larvae, pupae, male and female adults), which were collected from Fujian province. In total, we obtained approximately 90 Gb of data, from which 33,720 unigenes were assembled and 17,797 unigenes were annotated. We furtherly analyzed the differentially expressed genes (DGEs) across all stages, the largest number between the eggs and 1st instar larvae stage and gene expression varied significantly in different developmental stages. Furthermore, 4138 SSR genes and 114,977 SNP loci were screened from transcriptome data. This paper will be a foundation for further study towards improved integrated pest management strategies for this species.

## 1. Introduction

**Figure 1:**
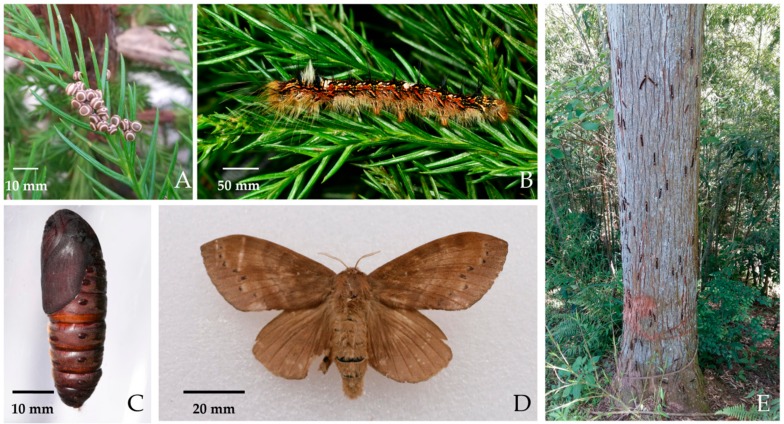
Four stages of *D. houi;* (**A**): Egg, (**B**): Larva, (**C**): Pupa, (**D**): Adult, (**E**): Pest outbreak.

The pine moth *Dendrolimus houi* Lajonquiere (Lepidoptera: Lasiocampidae) is a widely distributed and adaptable phytophagous pest, which seriously infests leaves of *Cryptomeria fortunei*, *Pinus yunnanensis*, *Pinus massoniana*, *Cupressus funebris*, *Platycladus orientalis* during its larval stage, causing thousands of hectares of dead and dying coniferous trees (Figure 1) in South China. Previous studies mostly focused on distribution, host range, biology, natural enemies and pest management approaches [1,2,3,4]. This pest develops on conifers mostly at high elevations and can have one or two generations per year depending on the local climate. Interestingly, it has a much longer development time, particularly in the larval stages, than other species of *Dendrolimus* in China, including *D*. *punctatus* Walker [5], *D*. *kikuchii* Matsumura [6], *D. superans* Butler [7], *D. spectabilis* Butler [8] and others. Over the approximately 150 d larval development time, the body length of larva grows from 7 mm (the 1st instar larva) to 116 mm (the final instar larva, 7st instar) and during this time there are significant changes in the caterpillar, including developing toxic setae. There is a tendency to overwinter as eggs or the 1st instar larvae. These stages have strong tolerance to low (above freezing) temperatures (under 5 °C) in the winter, while older larvae to final instar larvae and pupae have tolerance to high temperatures (above 35 °C) in the warmest summer months. Different stages of this pest have some special biological adaptations to ecological factors. Consequently, we inferred that a series of physiological changes and adaptations have taken place during the developmental process. However, little is known about development and regulatory mechanisms of *D. houi* at the molecular level. Transcriptomics and *de novo* transcriptome reconstruction is a robust method to characterize these mechanisms across developmental stages of *D. houi*.

Technically, the high-throughput next generation sequencing technology (NGS) has greatly promoted the application of insect transcriptomics [9,10,11,12,13,14], mostly focusing on their growth and development, classification, toxicology, interaction between insects and host plants and even non-coding RNA. For instance, transcriptomic data of *Adelphocoris suturalis* and *D. punctatus* across different stages was obtained using NGS techniques to define the gene expression related to the development of insects [15,16,17] and that of *Spodoptera frugiperda* was sequenced to reveal the mechanism of antivirus resistance [18]. In this study, four stages across all life cycle of *D. houi* were sampled to obtain transcriptome data and understand the gene expression associated with development, which will be very helpful to potentially reveal the gene function related to regulatory mechanism of development, phylogeny and evolution and the interaction between insects and other organisms.

## 2. Materials and Methods

### 2.1. Sample Feeding

Adults of *D. houi* were collected from Fuzhou, Fujian province, China. Eggs were collected after mating, larvae were reared with fresh *C*. *fortunei* twigs (26 ± 1 °C with a photoperiod 14: 10 (L: D) and relative humidity 70 ± 5%) [4]. Each development stage has three replicates and, to obtain a sufficient quantity of RNA, samples contained 120 eggs, 32 larvae of the 1st–3rd (L1-3) instar, 16 larvae of the 4th–5th (L4-5) instar, 8 larvae of the 6th–7th (L6-7) instar, 2 pupae and 1 male and 1 female adult respectively. For each sample, live specimens were put into liquid nitrogen and stored in a refrigerator at −80 °C until extraction.

### 2.2. RNA Isolation and Illumina Sequencing

Total RNA was extracted by TRIzoI Reagent (Invitrogen, Carlsbad, CA, USA), then the total RNA degradation and contamination was monitored on 1% agarose gels. The purity of RNA was determined by Nanodrop spectrophotometer (IMPLEN, Westlake Village, CA, USA), RNA concentration was measured using Qubit^®^ RNA Assay Kit in Qubit^®^2.0 Flurometer (Life Technologies, Carlsbad, CA, USA) and the integrity was accurately determined by Agilent 2100 (Agilent Technologies, Santa Clara, CA, USA). Sequencing libraries were generated using NEBNext^®^Ultra^TM^ RNA Library Prep Kit for Illumina^®^ (NEB, Ipswich, MA, USA), following the manufacturer’s recommendations and index codes were added to attribute sequences to each sample. Briefly, mRNA was purified from total RNA using poly-T oligo-attached magnetic beads. First strand cDNA was synthesized using random hexamer primer and reverse transcriptase (Invitrogen, Carlsbad, CA, USA). Second strand cDNA synthesis was subsequently performed using DNA polymerase I and RNase H. Then the high-throughput RNA-sequencing libraries were prepared, following Illumina’s protocols and were sequenced on the Illumina Hiseq 2000 platform (Illumina, San Diego, CA, USA).

### 2.3. De Novo Transcriptome Assembly and Annotation

In order to obtain high quality clean reads, the raw reads were filtered to remove adaptor fragments, reads containing unknown nucleotide “N” over 5% and empty tags. Trinity v2.5.1 (Broad Institute, Cambridge, MA, USA) with min-kmer-cov set to 2 by default and all other parameters set default [19] was used to assemble all reads from all stages for individual samples and the TGICL v2.1 clustering tool [20] was utilized to assemble all the unigenes with default parameters. Then, the unigenes were aligned with the Nr (non-redundant protein sequence), Swiss-Prot (a manually annotated, non-redundant protein sequence), COG (clusters of orthologous groups of proteins), KOG (clusters of protein homology), eggNOG 4.5 (orthologous groups of genes) and KEGG (kyoto encyclopedia of genes and genomes) databases by BLAST v2.2.31 with a cut-off e-valve of 10^−5^ [21] and GO (gene ontology) annotation was performed using Blast2GO v2.5 [22]. TransDecoder 5.0.0 (Broad Institute, Cambridge, MA, USA) was used to predict the amino acid sequence of unigene and the HMMER v3.1b2 software was used to search the Pfam database to get annotation information of the unigenes.

### 2.4. Sequencing and Analysis of Differential Gene Expression Profile

Based on FPKM (fragments per kilobase of transcript per million mapped reads) value, the levels of unigene expression were calculated and normalized. Differential expression analysis of sample groups was performed using the DESeq R package (1.10.1). DESeq provides statistical routines for determining differential expression in digital gene expression data using a model based on the negative binomial distribution, and the generally accepted Benjamini-Hochberg method [23] was adopted to correct the p-value of the original hypothesis tests and finally adopted it (False Discovery Rate, FDR). It was the key indicator of differential gene expression screening to reduce false positives resulting from independent statistical hypothesis testing of the expression values of a large number of genes [24]. We use “FDR ≤ 0.001 and the absolute value of Log2 ratio ≥ 1” as the threshold to judge the significance of the gene expression difference. Then, the specificity of gene expression variation across eggs-L1-3 instar larvae, L1-3-L4-5 instar larvae, L4-5-L6-7 instar larvae, L6-7 instar larvae-pupae and pupae-adults was analyzed respectively. The change and enrichment of differentially expressed genes at five different stages by functional category was performed using GO and KEGG pathways enrichment analysis. GO enrichment analysis, which were calculated the gene numbers for each term, determining the GO terms that were significantly enriched with DEGs by using a hypergeometric test [25]. The calculated *p*-value was subjected to correct, using the corrected *p*-value (q-value) ≤ 0.05 as a threshold. And KEGG pathway enrichment analyses with a q-value ≤ 0.05 were considered significantly enriched.

### 2.5. Microsatellite Identification

All assembled unigenes more than 1 kb in length were used to identify simple repeats using MISA. The reporting criteria are as follows: mono-nucleotide repeat threshold is ≥ 10, di-nucleotide repeat threshold is ≥ 6, trinucleotide to hexa-nucleotide repeat threshold is ≥ 5.

### 2.6. Single Nucleotide Polymorphism

The sequence alignment software STAR 2.6.0b was used to align the raw RNA-seq reads to the unigene sequence for each sample and GATK v3.2.2 (Broad Institute, Cambridge, MA, USA) was used following its best practices to identify single nucleotides polymorphism (SNP) sites.

## 3. Results

### 3.1. Sequencing and de Novo Transcriptome Assembly

In total, after filtering, 138,340,761 clean reads were obtained across the different development stage samples of *D. houi* with generally uniform coverage across samples (min of 20.7 M reads, max of 26.3 M reads, Table 1). The clean reads sequences were assembled by using Trinity (default parameters). A total of 65,149 contigs and 33,720 transcripts unigenes with a length ranging from 300 to 3000 bp were obtained. Among them, 22,012 unigenes were > 500 bp and 13,999 were > 1000 bp. The length of unigenes between 300 and 500 bp accounted for 34.71% (Table 2).

### 3.2. Predictive Protein Annotation

**Figure 2:**
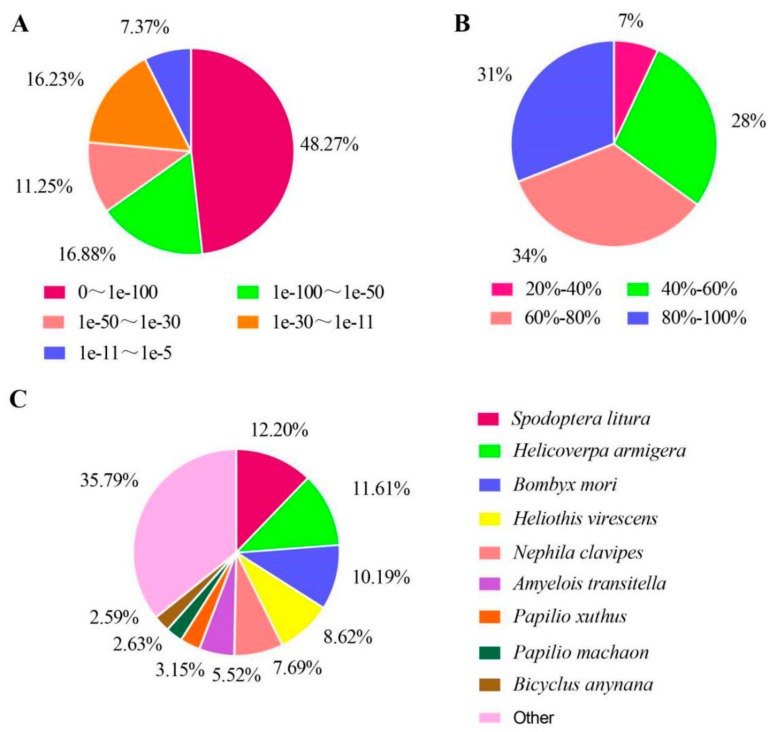
Pie-charts showing distributions from BLAST (Basic Local Alignment Search Tool) matches of *D. houi* transcriptome unigenes with respect to (**A**) (e-value distribution of unigenes of *D. houi*), (**B**) (Similarity distribution of unigenes of *D. houi*) and (**C**) (Species distribution of unigenes of *D. houi*).

A total of 17,797 unigenes with annotated information were obtained (Table 3), accounting for 52.78% of the total sequences, suggesting that some transcripts may be unique to this species or may be significantly variable from homologous transcripts in other species. The unigenes annotated in the Nr database, 34.85% having significant homology (< 1e^−5^–1e^−50^) (Figure 2A). The species similarity distributions showed that the majority of the matches were concentrated between 60% and 80% similarity (Figure 2B) and 4898 sequences had sequence similarity more than 80%. Matches to the Nr database showed the highest similarity to *Spodoptera litura*, accounting for 12.20%, followed by *Helicoverpa armigera*, *Bombyx mori*, *Heliothis virescens*, *Nephila clavipes*, *Amyelois transitella*, *Papilio xuthus*, *Papilio machaon*, *Bicyclus anynana* and others in decreasing order (Figure 2C).

### 3.3. Functional Classification and Metabolic Pathway Analysis of Unigenes

**Figure 3:**
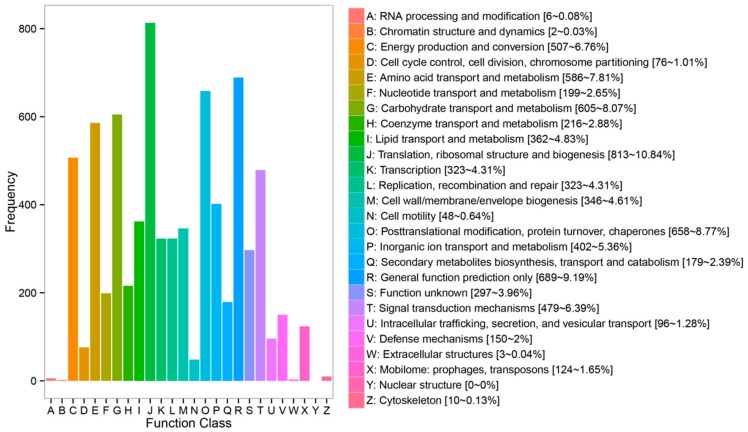
COG (clusters of orthologous groups of proteins) classification. The name (including number and percentage present) for each class definition is also provided: A: RNA processing and modification (6, 0.08%), B: Chromatin structure and dynamics (2, 0.03%), C: Energy production and conversion (507, 6.76%), D: Cell cycle control, cell division, chromosome partitioning (76, 1.01%), E: Amino acid transport and metabolism (586, 7.81%), F: Nucleotide transport and metabolism (199, 2.65%), G: Carbohydrate transport and metabolism (605, 8.07%), H: Coenzyme transport and metabolism (216, 2.88%), I: Lipid transport and metabolism (362, 4.83%), J: Translation, ribosomal structure and biogenesis (813, 10.84%), K: Transcription (323, 4.31%), L: Replication, recombination and repair (323, 4.31%), M: Cell wall/membrane/envelope biogenesis (346, 4.61%), N: Cell motility (48, 0.64%), O: Posttranslational modification, protein turnover, chaperones (658, 8.77%), P: Inorganic ion transport and metabolism (402, 5.36%), Q: Secondary metabolites biosynthesis, transport and catabolism (179, 2.39%), R: General function prediction only (689, 9.19%), S: Function unknown (297, 3.96%), T: Signal transduction mechanisms (479, 6.39%), U: Intracellular trafficking, secretion and vesicular transport (96, 1.28%), V: Defense mechanisms (150, 2%), W: Extracellular structures (3, 0.04%), X: Mobilome: prophages, transposons (124, 1.65%), Y: Nuclear structure (0, 0%), Z: Cytoskeleton (10, 0.13%).

In the COG database, the statistical results indicated that a total of 8436 protein sequences have been matched and divided into 26 categories. Most assignment within the biological functions category were assigned to “translation, ribosomal structure and biogenesis” (813/10.84%), “general function prediction” (689/9.19%), “posttranslational modification, protein turnover, chaperones” (658/8.77%), “carbohydrate transport and metabolism” (605/8.07%), “amino acid transport and metabolism” (586/7.81%), “energy production and conversion” (507/6.67%) and “signal transduction mechanism” (479/6.39%) (Figure 3).

**Figure 4:**
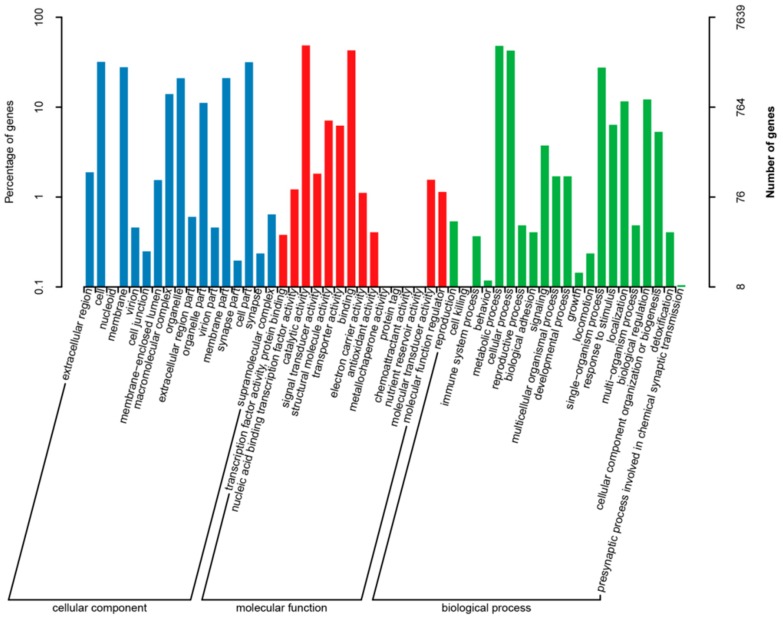
GO (gene ontology) classification. GO categories of “Cellular component “ (blue): extracellular region, cell, nucleoid, membrane, virion, cell junction, membrane-enclosed lumen, macromolecular complex, organelle, extracellular region part, organelle part, virion part, membrane part, synapse part, cell part, synapse, supramolecular complex; “Molecular function”(red): transcription factor activity, protein binding, nucleic acid binding transcription factor activity, catalytic activity, signal transducer activity, structural molecule activity, transporter activity, binding, electron carrier activity, antioxidant activity, metallochaperone activity, protein tag, chemoattractant activity, nutrient reservoir activity, molecular transducer activity, molecular function regulator; “Biological process “(green): reproduction, cell killing, immune system process, behavior, metabolic process, cellular process, reproductive process, biological adhesion, signaling, multicellular organismal process, developmental process, growth, locomotion, single-organism process, response to stimulus, localization, multi-organism process, biological regulation, cellular component organization or biogenesis, detoxification, presynaptic process involved in chemical synaptic transmission.

A total of 7639 genes have been annotated to GO terms inferred from BLAST results. 53 GO terms could be obtained according to the three categories of “biological process,” “molecular function” and “cell component” in the GO database. Most of them were concentrated in “biological process” (12,541), followed by “cell component” (8595) and “molecular function” (12,588). “Biological process” mainly included “metabolic process” (48.07%), “cell process” (42.61%), “single organism process” (27.57%), “bioregulation” (12.19%) and “locomotion” (11.6%). “Cell component” consists mainly of “cell” (31.9%), “cell partial” (31.63%), “membrane” (27.79%), “membrane partial” (21.05%), “organelle” (21%), “polymer complex” (13.98%) and “organelle partial” (11.15%). “Catalytic activity” (48.59%) and “binding” (42.85%) were the two categories with the majority of annotations in “molecular function.” (Figure 4).

Furthermore, metabolic pathways of all unigenes were analyzed using KEGG database, a total of 6230 genes were annotated to 218 KEGG pathways. The most representative pathways were ribosome (426/6.84%), oxidative phosphorylation (190/3.05%), carbon metabolism (160/2.57%), protein processing in endoplasmic reticulum (139/2.23%), RNA transport (138/2.22%), purine metabolism (129/2.07%) and splice (127/2.04%).

### 3.4. Comparison and Analysis of Transcriptome at Different Developmental Stages of D. Houi

**Figure 5:**
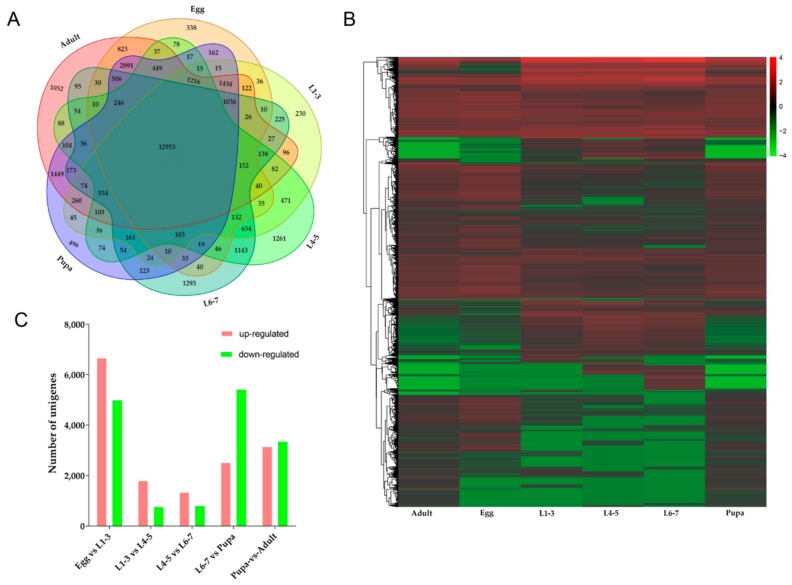
Comparison of transcriptome profiles of all developmental stages. (**A**): Venn diagram shown the unique and shared unigenes during *D. houi* development; (**B**): Heatmap of gene expression profiles; (**C**): The number of up-down regulated genes.

According to the Venn diagram, there were 338 unique genes at eggs stage, 230 unique genes from L1-3 instar larvae, 1261 unique genes from L4-5 instar larvae, 1293 unique genes from L6-7 instar larvae, 496 unique genes from pupae stage and 1052 unique genes at adults stage and 12,953 genes expressed across all stages, accounting for 45.1% of the total (Figure 5A). FPKM method was used to evaluate the gene expression level, five groups (eggs-L1-3 instar larvae, L1-3-L4-5 instar larvae, L4-5-L6-7 instar larvae, L6-7 instar larvae-pupae and pupae-adults) were compared and the genes with significant difference in expression were identified (FDR ≤ 0.001, Log2 ratio ≥ 1). In the heatmap, the gene expression profiles of different stages were significantly different and each stage was composed of a major cluster of highly expressed genes (Figure 5B). We found some embryogenesis genes such as vasa, mago nashi, easter, cactus, dorsal, VgR, maternal effect genes (bicoid, nanos), gap genes (hunchback, kruppel) and segment polarity genes (ftz) that were highly expressed during the egg stage. Epidermal protein and cuticle protein genes had high expression in larva and pupa stages. Chorion protein and chitin binding protein genes were identified in adult stage.

**Figure 6:**
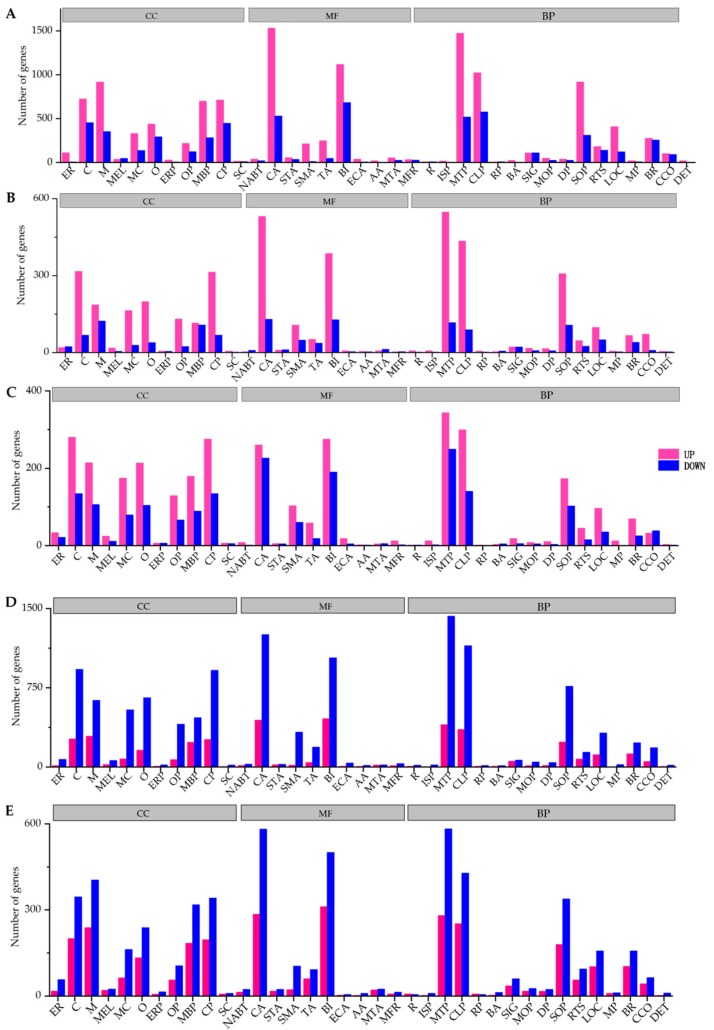
The representative GO terms enriched by differently expressed genes during different stages. (**A**): egg to L1-3 instar larva, (**B**): L1-3 to L4-5 instar larva, (**C**): L4-5 to L6-7 instar larva, (**D**): L6-7 instar larva to pupa, (**E**): pupa to adult. CC: cellular component, including extracellular region (ER), cell (C), membrane (M), membrane-enclosed lumen (MEL), macromolecular complex (MC), organelle (O), extracellular region part (ERP), organelle part (OP), membrane part (MBP), cell part (CP), supramolecular complex (SC); MF: molecular function, including nucleic acid binding transcription factor activity (NABT), catalytic activity (CA), signal transducer activity (STA), structural molecule activity (SMA), transporter activity (TA), binding (BI), electron carrier activity (ECA), antioxidant activity (AA), molecular transducer activity (MTA), molecular function regulator (MFR); BP: biological process, including reproduction (R), immune system process (ISP), metabolic process (MTP), cellular process (CLP), reproductive process (RP), biological adhesion (BA), signaling (SIG), multicellular organismal process (MOP), developmental process (DP), single-organism process (SOP), response to stimulus (RTS), localization (LOC), multi-organism process (MP), biological regulation (BR), cellular component organization or biogenesis (CCO), detoxification (DET).

**Figure 7:**
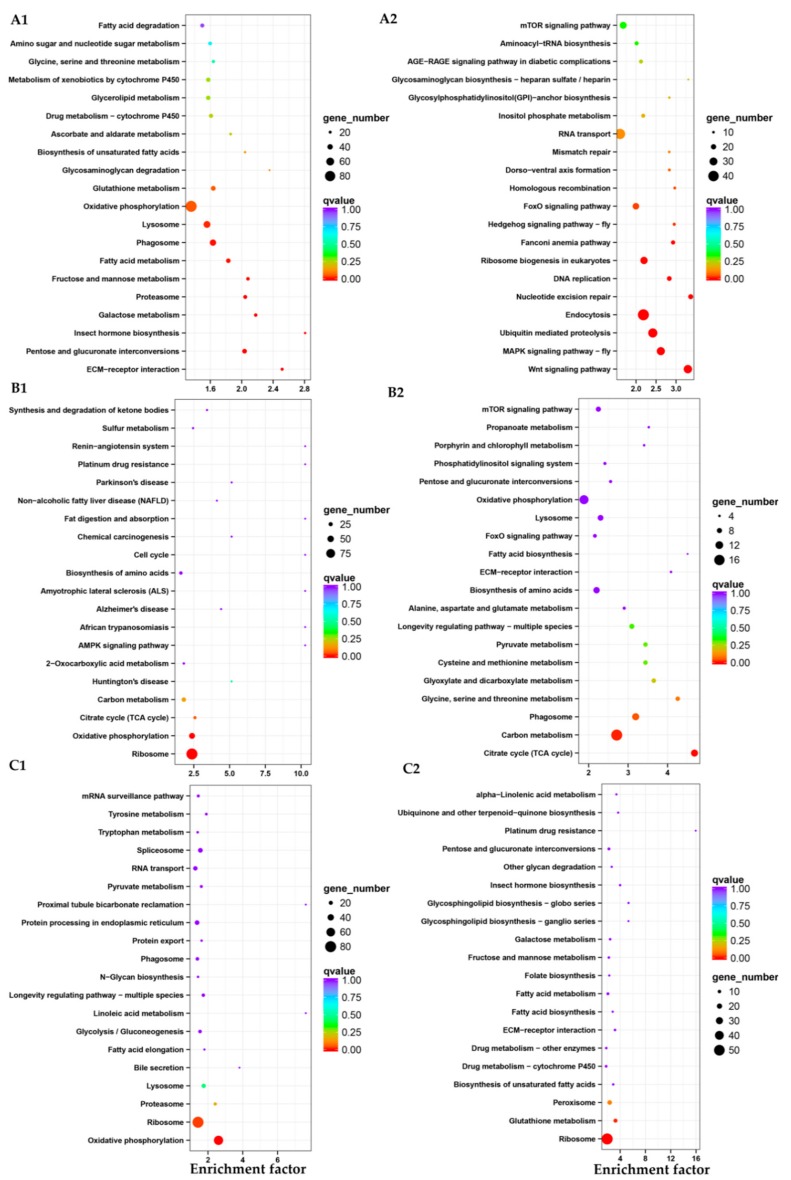
Enrichment and dispersion point map of differentially expressed gene of KEGG pathway. (**A1**–**C1**) means the up-regulated genes of KEGG pathway from eggs to L1-3 instar larvae stage, L1-3 to L4-5 instar larvae stage, L4-5 to L6-7 instar larvae stage, respectively; (**A2**–**C2**) means the down-regulated genes of KEGG pathway from eggs to L1-3 instar larvae stage, L1-3 to L4-5 instar larvae stage, L4-5 to L6-7 instar larvae stage, respectively. Each circle in the graph means a KEGG pathway, x-axis is the enrichment factor and y-axis means the name of the pathway.

During different stages from eggs to L1-3 instar larvae, the expression profiles of 11,662 genes were changed, among which 6669 genes were up-regulated and 4993 genes were down-regulated. From L1-3 to L4-5 instar larvae, 1804 genes were up-regulated and 766 genes were down-regulated. There were 1345 and 809 up-down regulated genes respectively from L4-5 to L6-7 instar larvae (Figure 5C). The most up-regulated genes from eggs to L1-3 instar larvae were *glycerol kinase*, *fatty acid-binding protein, trehalose transporter*, *sorbitol dehydrogenase*, *tyrosine-protein phosphatase 10D*, *cytochrome P450s*, *alpha-esterase 40 precursor*, *cuticle protein* and *NADH dehydrogenase subunit 5* and the most down-regulated genes were *vam6/Vps39*, *metaxin-1 isoform X2*, *kinesin-like protein KIF23*. Genes of *Glycoside hydrolase*, *lipA* and *epidermal retinol dehydrogenase* were up-regulated and *GMP reductase*, *fatty acid-binding protein* were down-regulated from L1-3 to L4-5 instar larvae. Comparison between L4-5 and L6-7 instar larval stage identified the genes of *heat shock protein*, *juvenile hormone*, *epidermis protein* (*cuticular protein RR-2 motif 63, larval cuticle protein LCP-22-like, cuticular protein hypothetical 8 precursor*), metabolism genes (*cytochrome P450s*, *carboxylesterases* and *glutathione S-transferases*) *antimicrobial peptide*, *endonuclease*, *collagenase* were up-regulated. The most down-regulated genes were histone genes (*histone H3.2, histone H1, late histone H2B.L4-like*), *vitellogenin receptor*, *protein synthesis protein*, *lipid metabolism* and *transport protein*, *yolk protein receptor*, *DNA synthesizer*. According to GO enrichment analysis (Figure 6), highly expressed genes were associated with metabolic process and catalytic activity (Figure 6A). In addition, KEGG enrichment analysis showed that during eggs to L1-3 instar larvae pathways related to lysosome, phagosome, fatty acid metabolism, fructose and mannose metabolism, proteasome, galactose metabolism, insect hormone biosynthesis, pentose and glucuronate interconversions, extracellular matrix (ECM)-receptor interaction were active (Figure 7A1). However, the up-regulated pathways have changed at the stages of L1-3-L4-5 and L4-5-L6-7 instar larvae (oxidative phosphorylation and ribosome, oxidative phosphorylation, respectively) (Figure 7B1,C1) and the down-regulated pathways also changed (Figure 7B2,C2).

**Figure 8:**
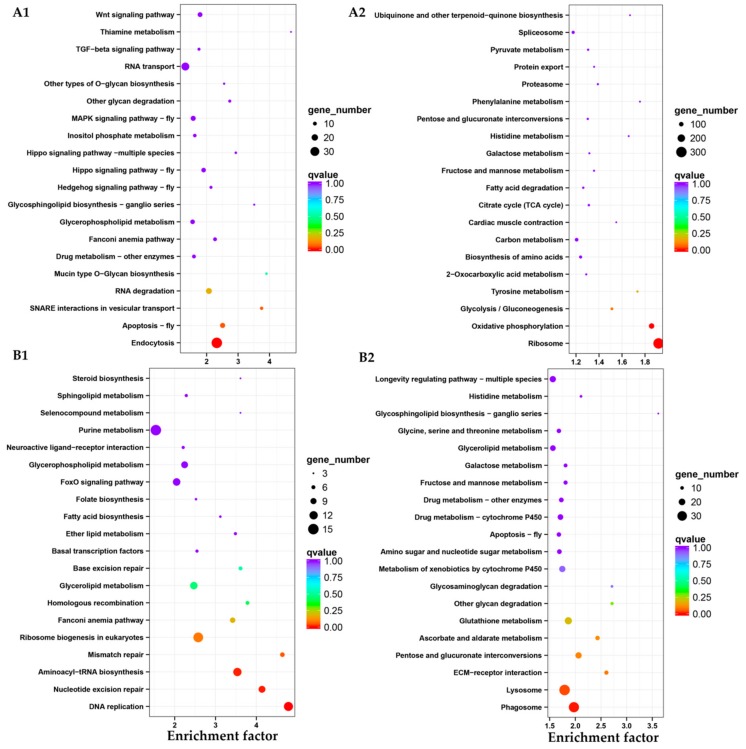
Enrichment and dispersion point map of differentially expressed gene of KEGG pathway. (**A1**,**B1**) means the up-regulated genes of KEGG pathway from L6-7 instar larval to pupal stage and pupae to adults stage; (**A2**,**B2**) means the down-regulated genes of KEGG pathway from L6-7 instar larvae to pupae stage and pupae to adults stage. Each circle in the graph means a KEGG pathway, x-axis is the enrichment factor and y-axis means the name of the pathway.

When the L6-7 instar larval stage transitioned to the pupal stage, there were 7941 genes expressed differently, including 2520 genes up-regulated and 5421 genes down-regulated (Figure 5C). The most up-regulated genes were *alpha-tocopherol transfer protein*, *inhibitor of growth protein*, *fatty acid synthase*, *heat shock protein*, *lysozyme*, *storage protein* and *insulin*, while the most down-regulated genes were *larval cutin*, *ion channel*, *glycerol kinase*, *cellulase*, *sensory protein*, *odorant binding protein*, *chitin* metabolism genes (*cytochrome P450*) and so on. In GO enrichment analysis (Figure 6D), it mainly focused on biological process, including metabolic process, cellular process and single-organism process. Then, it was catalytic activity, binding and structural molecule activity (molecular function), cell part, the composition of the whole membrane (cellular composition). The same as up-regulation process, the biological process was also dominant at down-regulation process. A little number of pathways were up-regulated, including endocytosis, apoptosis-fly and soluble NSF attachment protein receptor (SNARE) interactions in vesicular transport and two pathways were significantly down-regulated, including oxidative phosphorylation and ribosome (Figure 8A1,A2).

Finally, there were 6508 genes expressed differently, including 3152 genes up-regulated and 3356 genes down-regulated (Figure 5C) from pupa to adult stage. The most up-regulated genes were, *hydrolase*, *DNA synthesis*, *TFEB, structural protein*, *G1/S-specific cyclin-E*, *odorant-binding protein*, *neuropeptide receptor*, *chemosensory protein*, *multiple epidermal growth factor*, *transporter* and so on, while the most down-regulated genes were *toll-like receptor*, *hemolymph proteinase*, *juvenile hormone esterase*, *carboxypeptidase*, *lysozyme*, *lipase* and so on. Based on GO enrichment analysis (Figure 6E), it was mainly concentrated in biological processes, most of them were related to cell proliferation, such as reproduction, metabolic process, cellular process, reproductive process, biological adhesion, multicellular organismal process, developmental process and so forth. These results indicated that the main gene expression during adult stage was in the process of cell proliferation, which it could be inferred that the survival mode of *D. houi* during adult stage was mainly for the purpose of reproduction. However, in the process of down-regulation, molecular function was dominant, including catalytic activity, binding, structural molecule activity, transporter activity and so on. In KEGG enrichment analysis (Figure 8B1,B2), DNA replication, nucleotide excision repair and aminoacyl-tRNA biosynthesis were the most up-regulated, only pathway of phagosome was the most down-regulated.

### 3.5. SSR and SNP Analysis of D. Houi

**Figure 9:**
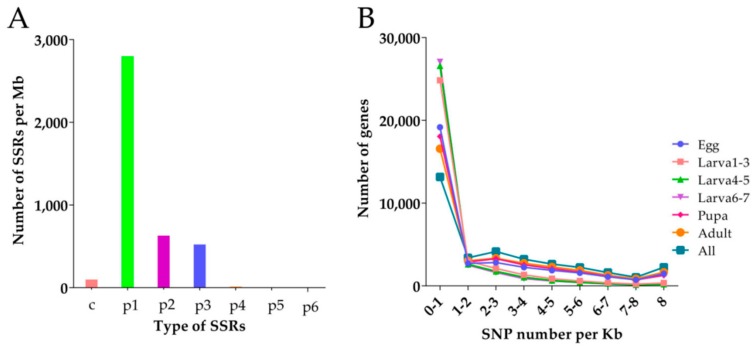
Density distribution. (**A**): SSR (Simple Sequence Repeats) density distribution, the X-axis is the type of SSR and the y-axis is the number of SSR of the corresponding type in each Mb sequence; (**B**): SNP (Single Nucleotide Polymorphism) density distribution, the x-axis is SNP density, that is, the number of SNP per Kb gene sequence and the y-axis is the number of genes with corresponding density.

A total of 4138 SSR (Simple Sequence Repeats) genes were searched from the transcriptome of *D. houi*, mono-nucleotide repeats were the most prevalent repeat type (Figure 9A), followed by di-nucleotide repeats (644), trinucleotide repeats (536) and compound SSR (113), tetra-nucleotide repeats (28), penta-nucleotide repeats (4) and hexa-nucleotide (1). Motif A accounted for the largest number of mono-nucleotide repeats and motif AT was the most numerous in the di-nucleotide repeats, potentially representing the poly (A) tail of mRNA. In SSR data, the number of repeats in the range of 5–9 repeats was the highest and the number of repeats more than 24 was less (Table 4). Meanwhile, 10,239 primer pairs were designed as part of the analysis that could be used to target these SSR regions.

There were 429,997 SNP loci during all stages (Table 5), including 217,530 homozygous SNPs and 212,467 heterozygous SNPs in the transcriptome of *D. houi*. The number of SNP loci during larval stage were the least (61,269) but at the adult stage were the largest (Figure 9B).

## 4. Discussion

In this study, we obtained 86.48 Gb clean data from samples across all life stages of *D. houi* by using high throughput sequencing, assembling to 33,720 unigenes of which a total of 17,797 unigenes were finally annotated. For sequences that had BLAST match in the Nr database, the most common and most homologous matches were to *S*. *litura*, accounting for 12.20% of the top hits. We further analyzed the differentially expressed genes at different growth stages of the insect. There were 26,111 differentially expressed genes in the DGE libraries. From egg to whole larva stage, the differentially expressed genes accounted for 53.14%, which indicated that the regulatory mechanism might be more complex compared to other stages. The difference of gene expression across different stages indicated regulation mechanism during different stages, which can be further explored in future research.

The egg and newly hatched larva stage of *D. houi* presented high tolerance to the low temperature stress in the winter *and* we found that some resistance and energy storage genes were up-regulated such as *glycerol kinase*, *fatty acid-binding protein, trehalose transporter*, *sorbitol dehydrogenase*, *cytochrome P450s*, *alpha-esterase 40 precursor* and NADH (Nicotinamide Adenine Dinucleotide) *dehydrogenase subunit 5* from the egg to L1-3 instar larval stage. However, some high expression genes have changed with the increase of larval instar, external temperature and self-growth metabolism, the resistance of the older larvae to high temperature and other unfavorable external factors also increased. For example, gene of *acid-binding protein* began to be down-regulated of L4-5 instar larva but genes of *heat shock protein* (HSP), *juvenile hormone*, *epidermis protein*, *detoxifying enzymes* and so on were up-regulated in older larvae. Previous studies have shown that insects can improve the cold resistance by producing small molecules of cold-resistant substances such as glycerol, trehalose, sorbitol, glucose and so forth [26,27,28]. And they also depend on some kinds of proteins such as HSPs to resist high temperature, the HSPs play an important role in improving the heat resistance of organisms [29,30,31]. The ability of cold and heat resistance of *D. houi* may be related to these high expression genes. Moreover, Togawa et al. [32] found that the *CPFL2-7* epidermis protein gene of *Anopheles gambiae* was highly expressed in the larval stage, which may be involved in the formation of larval epidermis. We also assumed that these highly expressed epidermis protein genes in the larval stage might be involved in the epidermis formation of *D. houi* larvae. Interestingly, the up-regulation of *juvenile hormone* genes within larvae were relatively abundant, partially the long development duration associated with having seven instars of larvae, possibly playing a role in maintaining a long larval stage with many instars [33,34,35]. Furthermore, the expressed gene level of related detoxification metabolic enzymes was also high in the process of larval feeding, because detoxification enzymes (*cytochrome P450s*, *carboxylesterases* and *glutathione S-transferases*) were in vivo are needed to participate in the metabolic decomposition of secondary substances within host plants or some pesticides [9,36,37,38,39]. The up-regulation of these genes provided help for them to overcome the adverse environmental factors.

During the larval and pupal stage, there were differentially expressed 7941 genes, the up-regulated genes accounted for only 31.73% and the down-regulated genes reached 68.27%. Additionally, the gene expression levels of *fatty acid synthase*, *storage protein* and other genes were relatively high during this stage. The *fatty acid synthase* and *storage protein* was synthesized in the fat of feeding larvae and released into the hemolymph and reached the peak at the end of larval stage, which provided protein and amino acids for development of pupae and organs or tissue formation of adults, playing an important regulatory role for the storage of amino acids inside insects for normal metabolism [40,41,42]. Moreover, lysozyme was significantly up-regulated, which will be very helpful to overcome external adverse factors and provide favorable conditions for its autoimmunity [43,44,45].

From pupae to adults, there were 6508 differentially expressed genes and the number of up- regulated genes were close to down-regulated genes. In the process of adult emergence from pupal stage, the transcription factor TFEB (transcription factor EB) was relatively enriched. Sardiello et al. found that the synthesis and function of lysosome are regulated by a gene network and the coordinated expression of these genes is controlled by transcription factor *TFEB*. And *TFEB* itself can be activated during lysosomal dysfunction, regulated the abundance of lysosomes in cells and its ability to degrade complex molecules [46], which played an important role in the process of apoptosis and self-digestion of its own cells [44]. Furthermore, the expression of structural protein genes was up-regulated, which played an important role in the formation of tissue and organs as well as external morphological structure in the process of adults [47,48]. In addition, high expression of chemosensory genes is beneficial to adults mating, oviposition and other behaviors, which rely on sensitive olfactory mechanisms [10,49].

Technically, molecular marker is an important method for population genetics research [50,51]. In this study, 4138 SSR genes and 114,977 SNP loci were identified from the transcriptome of *D. houi*, mono-nucleotide repeat is the main type of SSR, accounting for the largest proportion, the trinonucleotide repeat was the most common type, which is similar to results of some previous research on *Epacromius coerulipes* [52], *Bactrocera dorsalis* [53]. Thousands of primer pairs were designed by transcriptome data, which greatly saved the time for primer design, although the validation assay of designed primers have not yet been performed, which provided a certain basis for the further study of genetic evolution, the SSR and SNP genes screened in this study will lay a foundation for the study of population genetics of this species at the same time, the study of differentially expressed genes will provides a new direction and method to formulate more reasonable integrated pest management. Further, we plan to further evaluate mechanisms for adaptation in future experiments, to evaluate how this species is able to adapt to extreme environments (e.g., temperature).

The pine moth *D. houi* is extremely harmful when it exhibits outbreaks, particularly in Yunnan and Fujian provinces. However, it has different generations in these two regions, which may be related to its environmental factors. The Yunnan population of *D. houi* perches and survives at high altitude all the year round, consequently, those caterpillars might be induced to strengthen their cold tolerance because they were usually exposed to longer period, higher frequency and stronger intensity of low temperature stress than Fujian population. Furtherly, this impact might also be partially induced by hosts of Yunnan population, *P. yunnanensis*, which prefer an area with high altitude from 2000 to 3200 m, while Fujian population of *D. houi* infests *C. fortunei*, which are located in the area with altitude of 400–2000 m. Interestingly, some previous research has shown that different host plants provided different nutrients and the insects feed on different hosts before overwintering, which affects the composition and accumulation of cold-resistant substances within their bodies, leading to differences in cold-resistant properties [26,54]; and *P. yunnanensis* was proved to be one of the most cold-resistant species hosts (grade I) [55]. On the contrary, the Fujian population survives under the longer period, higher frequency and stronger intensity of high temperature stress in the summer, which might strengthen their adaptation of older larvae or pupae to heat stress. Obviously, these two populations must be regulated by both temperature stress and host difference, which may lead to differential profiles of gene expression. We assumed these results will provide a theoretical basis for more targeted pest control.

## 5. Conclusions

In this study, four stages across all life cycle of *D. houi* were sampled to obtain transcriptome data and understand the gene expression associated with development, which will be very helpful to potentially reveal the gene function related to regulatory mechanism of development, phylogeny and evolution and the interaction between insects and other organisms. This paper will be a foundation for further study towards improved integrated pest management strategies for this species.

## Figures and Tables

**Table 1:** Summary of output statistics from selected *D. houi* developmental stages.

Sample	Clean Data	Raw Data	GC (%)	Q20 (%)
Adult	22,239,714	6,659,218,426	42.01	95.8
Egg	25,298,693	7,572,136,450	38.5	95.94
1st–3rd instar larva	20,748,014	6,211,582,592	51.25	96.21
4th–5th instar larva	22,182,643	6,635,257,832	53.01	96.42
6th–7th instar larva	26,275,877	7,872,265,062	50.66	96.04
Pupa	21,595,820	6,470,712,932	43.97	96.42

**Table 2:** Result of assembly from transcriptome of *D. houi.*

Sequence Length (bp)	Transcript Number	Unigene Number
300–500	15,369(23.59%)	11,705(34.71%)
500–1000	15,563(23.89%)	8014(23.77%)
1000–2000	16,025(24.60%)	6552(19.43%)
2000+	18,189(27.92%)	7446(22.08%)
Total Number	65,149	33,720
Total Length (bp)	108,319,868	47,972,049
N50 Length (bp)	2659	2534
Mean Length (bp)	1662.65	1422.66

**Table 3:** Statistical results of unigene annotation of *D. houi.*

Anno_Database	Annotated_Number	300≤ Length <1000	Length ≥1000
COG_Annotation	8435	4094	4341
GO_Annotation	7639	2400	5239
KEGG_Annotation	6230	1653	4577
KOG_Annotation	9597	2569	7028
Pfam_Annotation	13,459	4880	8579
Swiss-Prot_Annotation	8618	2216	6402
eggNOG_Annotation	15,310	5631	9679
nr_Annotation	15,911	5693	10,218
All_Annotated	17,797	7495	10,302

**Table 4:** Distribution of the SSR motifs in *D. houi* transcriptome.

SSR Type	Repeat Motif	Number	Frequency
Mono-nucleotide	A/T/G/C	2812	67.96%
Di-nucleotide	AT/GT/TA/TC Other types	644	15.56%
Trinucleotide	GTG/GTC/GAA/GAT/AAT/CGG/TTC/TAT Other types	536	12.95%
Tetra-nucleotide	ATTC/AGGA/GTAT/TCAC/TTAC Other types	28	0.68%
Penta-nucleotide	ATATC/CTCTG/GTACA/AAATG	4	0.097%
Hexa-nucleotide	TTCTCC	1	0.023%
Compound SSR	(A)10(AT)7 Other types	113	2.73%
Total		4138	100%

**Table 5:** SNP number statistics.

Samples	HomoSNP	HeteSNP	AllSNP
Egg	47,721	67,256	114,977
1st–3rd instar larva	35,301	25,968	61,269
4th–5th instar larva	30,498	17,964	48,462
6th–7th instar larva	31,420	12,572	43,992
Pupa	60,348	61,627	121,975
Adult	74,160	57,616	131,776
